# Effect of caffeine ingestion on anaerobic capacity quantified by different methods

**DOI:** 10.1371/journal.pone.0179457

**Published:** 2017-06-15

**Authors:** Lucyana Arcoverde, Rodrigo Silveira, Fabiano Tomazini, André Sansonio, Romulo Bertuzzi, Adriano Eduardo Lima-Silva, Victor Amorim Andrade-Souza

**Affiliations:** 1 Sport Science Research Group, Department of Physical Education and Sports Science, Academic Center of Vitoria, Federal University of Pernambuco, Vitória de Santo Antão, Pernambuco, Brazil; 2 Endurance Performance Research Group, School of Physical Education and Sport, University of São Paulo, São Paulo, São Paulo, Brazil; 3 Human Performance Research Group, Technological Federal University of Parana, Parana, Brazil; Victoria University, AUSTRALIA

## Abstract

We investigated whether caffeine ingestion before submaximal exercise bouts would affect supramaximal oxygen demand and maximal accumulated oxygen deficit (MAOD), and if caffeine-induced improvement on the anaerobic capacity (AC) could be detected by different methods. Nine men took part in several submaximal and supramaximal exercise bouts one hour after ingesting caffeine (5 mg·kg^-1^) or placebo. The AC was estimated by MAOD, alternative MAOD, critical power, and gross efficiency methods. Caffeine had no effect on exercise endurance during the supramaximal bout (caffeine: 131.3 ± 21.9 and placebo: 130.8 ± 20.8 s, *P* = 0.80). Caffeine ingestion before submaximal trials did not affect supramaximal oxygen demand and MAOD compared to placebo (7.88 ± 1.56 L and 65.80 ± 16.06 kJ *vs*. 7.89 ± 1.30 L and 62.85 ± 13.67 kJ, *P* = 0.99). Additionally, MAOD was similar between caffeine and placebo when supramaximal oxygen demand was estimated without caffeine effects during submaximal bouts (67.02 ± 16.36 and 62.85 ± 13.67 kJ, *P* = 0.41) or when estimated by alternative MAOD (56.61 ± 8.49 and 56.87 ± 9.76 kJ, *P* = 0.91). The AC estimated by gross efficiency was also similar between caffeine and placebo (21.80 ± 3.09 and 20.94 ± 2.67 kJ, *P* = 0.15), but was lower in caffeine when estimated by critical power method (16.2 ± 2.6 *vs*. 19.3 ± 3.5 kJ, *P* = 0.03). In conclusion, caffeine ingestion before submaximal bouts did not affect supramaximal oxygen demand and consequently MAOD. Otherwise, caffeine seems to have no clear positive effect on AC.

## Introduction

The maximum amount of ATP that can be resynthesized by the breakdown of intramuscular ATP, phosphocreatine and muscle glycogen during a short-duration maximal exercise is defined as anaerobic capacity (AC) [1–5]. It is assumed that the total energy available from anaerobic resources is finite [5]. The AC is an important parameter for measuring athletic performance during short-distance sports such as 500–4,000 m cycling time trials [6]. While there is no “gold-standard” method to estimate AC, it has been consensually assessed by the maximal accumulated oxygen deficit (MAOD) [4]. The MAOD is calculated by the difference between the accumulated oxygen uptake during an exhaustive supramaximal test, and the corresponding predicted oxygen demand estimated from several submaximal exercise bouts [1, 3].

A growing number of studies tested the effects of ergogenic agents on AC using the MAOD approach [2, 7–9]. Caffeine has been considered one of the most promising candidates influencing AC [2, 7, 10, 11]. The mechanism by which caffeine increases AC is poorly understood, but it has been suggested, although never tested in humans, that caffeine might antagonistically bind adenosine receptors in the muscle and withdraw adenosine-mediated inhibition of phosphofructokinase, which could increase the anaerobic glycolysis [12]. Recent evidence has also suggested that caffeine in physiological concentrations (~40 μmol·L^-1^) may improve calcium release from the sarcoplasmic reticulum during muscle contraction [13], decreasing the low-frequency fatigue [13, 14]. Caffeine might also act on the central nervous system increasing motivational drive and neuromuscular excitability and function, which, in turn, could result in a lowered rating of perceived exertion for a given workload [15, 16]. Additionally, caffeine attenuates muscle sensory signals to the brain and decreases the threshold of activation of motor neurons [17]. These peripheral and central effects could all influence AC. In fact, Doherty [2] and Bell et al. [7] observed an increase in MAOD after ingestion of 5 mg·kg^-1^ body mass of caffeine. However, in their studies, the submaximal exercise bouts to estimate supramaximal oxygen demand were performed without caffeine intake. Some studies have shown that caffeine does not affect VO_2_ in submaximal exercise [18, 19]; one study, however, has shown a reduced slow component of VO_2_ in intense exercise [20]. The development of a VO_2_ slow component at exercise intensities above the gas exchange threshold (GET) is the most likely cause affecting submaximal VO_2_-power output linearity. The use of submaximal exercise bouts above GET increases the slope of the VO_2_-power output curve in both untrained (~ 6%) and trained (~ 14%) men [21]. As some of the submaximal bouts used to estimate the supramaximal demand of MAOD develop a slow component of VO_2_, any effect of caffeine on the reduction of the slow component of VO_2_ would reduce the slope of the VO_2_-power output curve and ultimately the estimated oxygen demand [4]. Thus, a possible effect of caffeine on the submaximal VO_2_ response affecting the supramaximal oxygen demand should be taken into account.

There is another concern that has discouraged the use of MAOD in a practical routine. Exercisers are required to visit the laboratory several times to complete submaximal exercise bouts [1]. This is a very time-consuming process and consequently, other less time-demanding methods would be very appealing from a practical standpoint. The most common methodologies replacing MAOD are the W' parameter derived from the critical power (CP) concept, the deduction of the aerobic contribution from the external power output by calculating gross efficiency (GE), and the so-called alternative MAOD (MAOD_ALT_) [1, 22, 23]. This last one corresponds to the summation of the fast component of excess post-exercise oxygen consumption (EPOC_fast_) and an O_2_ equivalent from blood lactate accumulation [1]. This approach assumes that the EPOC_fast_ may correspond to the total energy used to resynthesize high-energy phosphate stores (i.e., alactic component) [24], while the O_2_ equivalent from blood lactate accumulation ([La^-^]) may represent the glycolytic energy cost (i.e., lactic component) [25]. A recent study has pointed out that caffeine improves supramaximal exercise endurance but not MAOD_ALT_, suggesting that either MAOD_ALT_ is not sensitive enough to detect changes in AC with caffeine or that the potential effect of caffeine during supramaximal exercise is a different fatigue-related process [26]. Therefore, studies testing caffeine effects using different approaches to estimate AC are required to provide insights as to whether caffeine-induced improvement on AC can be equally detected by these different methods.

The purposes of the present study were to examine whether caffeine ingestion during submaximal exercise bouts would affect the predicted supramaximal oxygen demand and MAOD, and to determine the sensitivity of the various methods of estimating AC for the detection of a potential caffeine effect. Our first hypothesis was that caffeine ingestion before submaximal bouts would reduce the predicted supramaximal oxygen demand, and consequently reduce the estimated MAOD. Our second hypothesis was that the different methods of estimating AC are equally sensitive for the detection of any caffeine influence.

## Materials and methods

### Participants

Nine recreationally active men participated in this study. The baseline demographic characteristics of the participants are provided in Table 1. Participants were recruited between July 2015 and December 2015 via online advertisements and leaflets at the University Campus. Because of the lack of data regarding the effects of caffeine on all methods of estimating AC, the required sample size was estimated using the data reported in Doherty [2] and Simmonds et al. [27] of the effect of caffeine on MAOD. With an alpha of 0.05 and a desired power of 0.80, the total effective sample size necessary to achieve statistical significance was estimated to be seven participants. However, assuming that 20% might drop out during the data collection, the sample size was increased to nine participants. Fourteen participants were approached, but only nine joined to the study. All participants who started the study completed all experimental trials. Participants signed a consent form agreeing to participate in the study, which had been approved by the ethics committee of the Federal University of Pernambuco (registration number 46017114.5.0000.5208).

**Table 1:** The baseline demographic characteristics of the study sample (mean ± SD).

Parameter	Mean ± SD
Age (years)	26.8 ± 5.9
Sex	Male
Nationality	Brazilian
Body mass (kg)	74.1 ± 7.0
Height (m)	1.73 ± 0.09
VO_2_peak (L·min^-1^)	2.99 ± 0.39
VO_2_peak (mL·kg^-1^·min^-1^)	40.6 ± 5.8

VO_2_peak, peak oxygen uptake.

### Design

In the first visit, the participants underwent a preliminary incremental exercise test on a cycloergometer (Ergo-Fit 167, Pirmasens, Germany). Then, the participants visited the laboratory 10 times, at least 48 h apart. This was a double-blind, crossover, randomized and balanced Latin Square counterbalanced measure design. The randomization was performed using software available at http://www.randomized.org. One hour before each experimental session, the participant ingested a capsule containing 5 mg·kg^-1^ body mass of caffeine or cellulose (placebo). Previous findings have suggested that plasma caffeine levels rise to ~ 40 μmol·L^-1^, peaking at 60–90 min after ingestion when caffeine doses are administrated in capsules containing 5 mg·kg^-1^ body mass [28]. The participants were asked to register all food and beverages consumed during the 24 h preceding the first experimental trial and repeat this before the subsequent trials. They were asked to not consume food containing caffeine and to refrain from any exercise 24 h before each test. Each trial session was performed at the same time of day for each participant to avoid circadian variability.

### Incremental test

After the participants had undergone a 3-min warm-up at 50 W, the power output was increased by 25 W·min^-1^ until volitional exhaustion (cadence between 70 and 80 rpm). Exhaustion was assumed when the participants were unable to maintain cadence above 70 rpm. VO_2_, VCO_2_ and ventilation were measured breath-by-breath throughout the test using an automatic gases analyzer (Cortex, Metalyzer 3B^®^, Saxony, Leipzig, Germany). Before each test, the gases analyzer was calibrated using ambient air, known O_2_ and CO_2_ concentrations and a 3-L syringe. The VO_2_peak was determined from the average VO_2_ during the last 30 s of the test. Two investigators identified the gas exchange threshold (GET) from a cluster of ventilatory and metabolic measurements: 1) the first disproportionate increase in VCO_2_ from visual inspection of individual plots of VCO_2_ *vs*. VO_2_; 2) an increase in VE/VO_2_ without increase in VE/VCO_2_; and 3) an increase in end-tidal O_2_ pressure with no fall in end-tidal CO_2_ pressure [29].

### Experimental trials

Seven constant-load tests were performed to satisfy the mathematical procedures of each method for estimating AC. The exercise intensities were based on percentage of VO_2_peak and delta (Δ) difference between the GET and VO_2_peak [30]: Δ10 (GET + 10% Δ), Δ20 (GET + 20% Δ), Δ40 (GET + 40% Δ), Δ50 (GET + 50% Δ), Δ80 (GET + 80% Δ), and 100 and 120% of VO_2_peak. We opted to determine submaximal bouts in relation to GET and VO_2_peak rather than in a percentage of VO_2_peak to make metabolic stress and VO_2_ response less variable among participants and guarantee that VO_2_ slow component would appear [31, 32].

**Fig 1:**
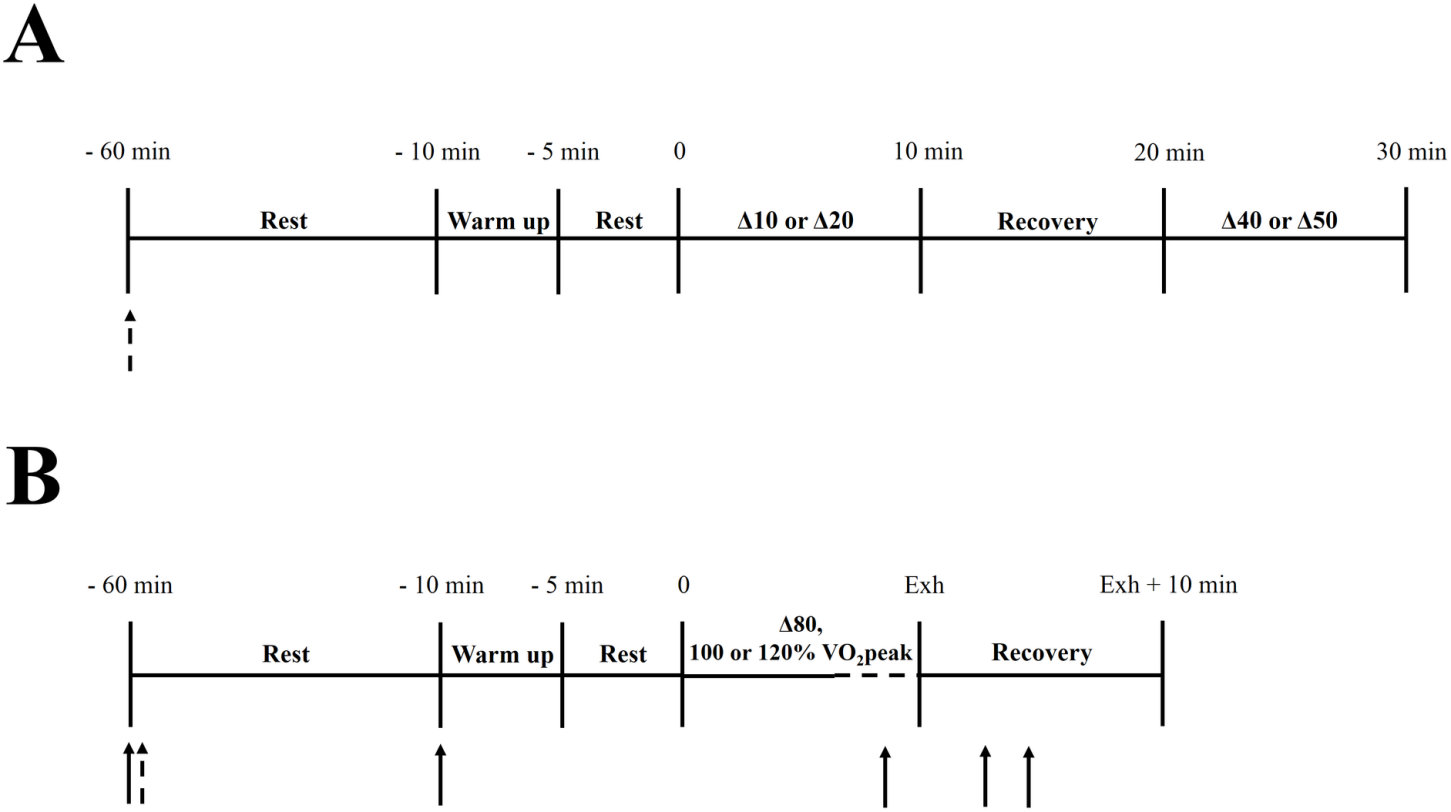
Experimental procedures. Dashed arrows indicate caffeine or placebo ingestion. Continuous arrows indicate blood sample collection (only at 120% VO_2_peak). Δ10, Δ20, Δ40, Δ50 and Δ80 represent the power output at gas exchange threshold (GET) plus the power output corresponding to the difference between GET and peak oxygen uptake (VO_2_peak).

Participants followed two different exercise protocols. In protocol 1 (Fig 1A), the participants arrived at the laboratory and ingested caffeine or placebo (-60 min). Fifty minutes later, participants began a 5-min warm-up at 90% GET. After 5 min rest, participants cycled for 10 minutes at Δ10 or Δ20, followed by 10-min of passive recovery, and cycled for 10 more minutes at Δ40 or Δ50. Protocol 2 (Fig 1B) followed a similar timeline, except that only one experimental bout (Δ80, 100 or 120% VO_2_peak) was performed and participants cycled until exhaustion. Gas exchanges were recorded breath-by-breath during the entire exercise in all trials. In addition, in order to calculate MAOD_ALT_ (see below), gas exchanges were measured during the 10-min recovery and capillary blood samples were collected from the earlobe (40 μL) before and at 1, 3 and 5 min after 120% VO_2_peak bout. Blood samples were immediately centrifuged at 3000 rpm for 10 min and plasma [La^-^] determined with a spectrophotometer (Quimis, Q798U2V5, Sao Paulo, Brazil) using commercial kits (Biotecnica, Varginha, Brazil).

Protocol 1 was repeated in two occasions to complete all workloads. These two runs of protocol 1 and three runs of protocol 2 were duplicated to fulfill the caffeine and placebo treatments, totaling 10 visits and seven different workloads performed under each experimental condition.

### Analysis

The VO_2_ corresponding to each submaximal exercise bout (Δ10, Δ20, Δ40 and Δ50) was defined as the average over the last 30 s [1]. To avoid any effect of exhaustion on VO_2_, the VO_2_ value corresponding to Δ80 was calculated at isotime. Isotime was defined as the shortest time to exhaustion recorded between caffeine and placebo conditions. These values were plotted against power output and supramaximal oxygen demand estimated by linear extrapolation. The VO_2_ measured during the 120% VO_2_peak bout was integrated over time to obtain the accumulated VO_2_ and MAOD calculated as the difference between the estimated and the accumulated VO_2_ [3].

MAOD under caffeine condition was calculated by using oxygen demand estimated by submaximal tests (Δ10, Δ20, Δ40, Δ50 and Δ80), either under the influence of caffeine (MAOD_CAF-CAF_) or with no caffeine (MAOD_CAF-PLA_). The MAOD under the placebo condition was calculated by using oxygen demand estimated without caffeine influence (MAOD_PLA-PLA_). Absolute MAOD values were reduced by 10% to correct for the contribution of body oxygen stores [3].

For MAOD_ALT_, the 10-min VO_2_ off-transient after the supramaximal exercise test was fitted using Eq 1 (Origin 8.5, Microcal, Massachusetts, USA):

$${VO}_{2(t)}={VO}_{2base}+A_{1}(e^{-(t-\delta )/\tau 1})+A_{2}[e^{-(t-\delta )/\tau 2}]$$ (1)

Where VO_2_ (*t*) is the oxygen uptake at time *t*, VO_2base_ is the oxygen uptake at the end of the recovery, A is the amplitude, δ is the time delay, τ is the time constant, and 1 and 2 denote the fast and slow components, respectively.

The alactic component of the MAOD_ALT_ was obtained by multiplying A_1_ by τ_1_ [1, 24, 33, 34]. The lactic component was estimated considering an accumulation of 1 mM of plasma [La^-^] equivalent to 3 ml O_2_·kg^-1^ body mass [25]. Plasma lactate accumulation was calculated as the difference between the highest plasma [La^-^] value after the supramaximal exercise ([La^-^]_peak_) and the [La^-^] at rest [1, 33–35]. For both MAODs, an energy equivalent of 20.9 kJ·LO_2_^−1^ was used.

Three CP models were initially tested (Eqs 2, 3 and 4) [36]:

Nonlinear:t=W′/(P−CP) (2)

Linear:W=W′+CP∙t (3)

Linearinverseoftime:P=(W′/t)+CP (4)

Where: *t* is the time to exhaustion, *W* is the total work performed, *W´* is the constant curvature of hyperbole power-time, *P* is the power output and CP critical power.

The Δ80, 100 and 120% VO_2_peak and their respective times to exhaustion were fitted with these equations. The model with the lowest standard error of the estimate for *W´* was chosen for further analysis [37].

For the GE method, the metabolic power during the warm-up period was calculated using Eq 5 [38]:

$$\mathrm{MetP}\;(W)={VO}_{2}(L∙{min}^{-1})\times [(4940\;RER+16040)/60]$$ (5)

Where MetP is metabolic power and RER is the respiratory exchange ratio (VCO_2_/VO_2_). VO_2_ and RER were calculated as the average of the last 30 s of the warm-up.

Gross efficiency was determined by dividing the external power output from the warm-up period by the calculated MetP. Then, MetP at 120% VO_2_peak test was estimated assuming a RER equal to 1.00 [39]. The aerobic mechanical power during the supramaximal exercise was calculated by multiplying the GE by the calculated MetP, assuming that GE remains stable after the GET [40]. The anaerobic mechanical power was calculated as the difference between external power output and aerobic mechanical power, and then integrated over the time to obtain the total anaerobic work, which is analogous to AC [23].

### Statistical analysis

Variables were tested for normality using the Kolmogorov-Smirnov test. One-way repeated measure ANOVA was used to compare the MAOD_PLA-PLA_, MAOD_CAF-PLA_ and MAOD_CAF-CAF_. Because both MAOD methods result in a “metabolic” estimate of AC, while CP and GE methods result in a “mechanical” estimate of AC, they are not directly comparable. Thus, the separated two-way repeated measure ANOVA was used to determine the effect of the method and condition between MAOD *vs*. MAOD_ALT_ and CP *vs*. GE. The MAOD_CAF-PLA_ was the method chosen for comparison with the MAOD_ALT_ [2, 7]. Two-way repeated measure ANOVA was also used to determine both the effect of exercise intensity and condition on time to exhaustion and submaximal VO_2_ and the effect of time and condition on plasma [La^-^] response during the supramaximal bout. When required, the Bonferroni’s post-hoc test was used to locate the difference. A paired *t* test was used to compare any other variables between caffeine and placebo. Significance was accepted when *P* < 0.05. Statistical analysis was performed using the Statistical Package for Social Sciences version 17.0 (SPSS Inc., Chicago, IL, USA).

## Results

The GET was identified at 102.5 ± 24.6 W. Maximal power output reached during the incremental test was 261.1 ± 26.1 W and VO_2_peak was 2.99 ± 0.39 L·min^-1^. There was a main effect of intensity, condition and interaction for time to exhaustion (*P* < 0.01). Compared to placebo, caffeine increased (*P* < 0.01) time to exhaustion at Δ80 (298.3 ± 71.3 *vs*. 400.0 ± 87.4 s, respectively), but not at 100% VO_2_peak (207.7 ± 37.7 *vs*. 233.6 ± 27.9 s, *P* = 0.64, respectively) and 120% VO_2_peak (130.8 ± 20.8 *vs*. 131.3 ± 21.9 s, *P* = 0.99, respectively).

**Fig 2:**
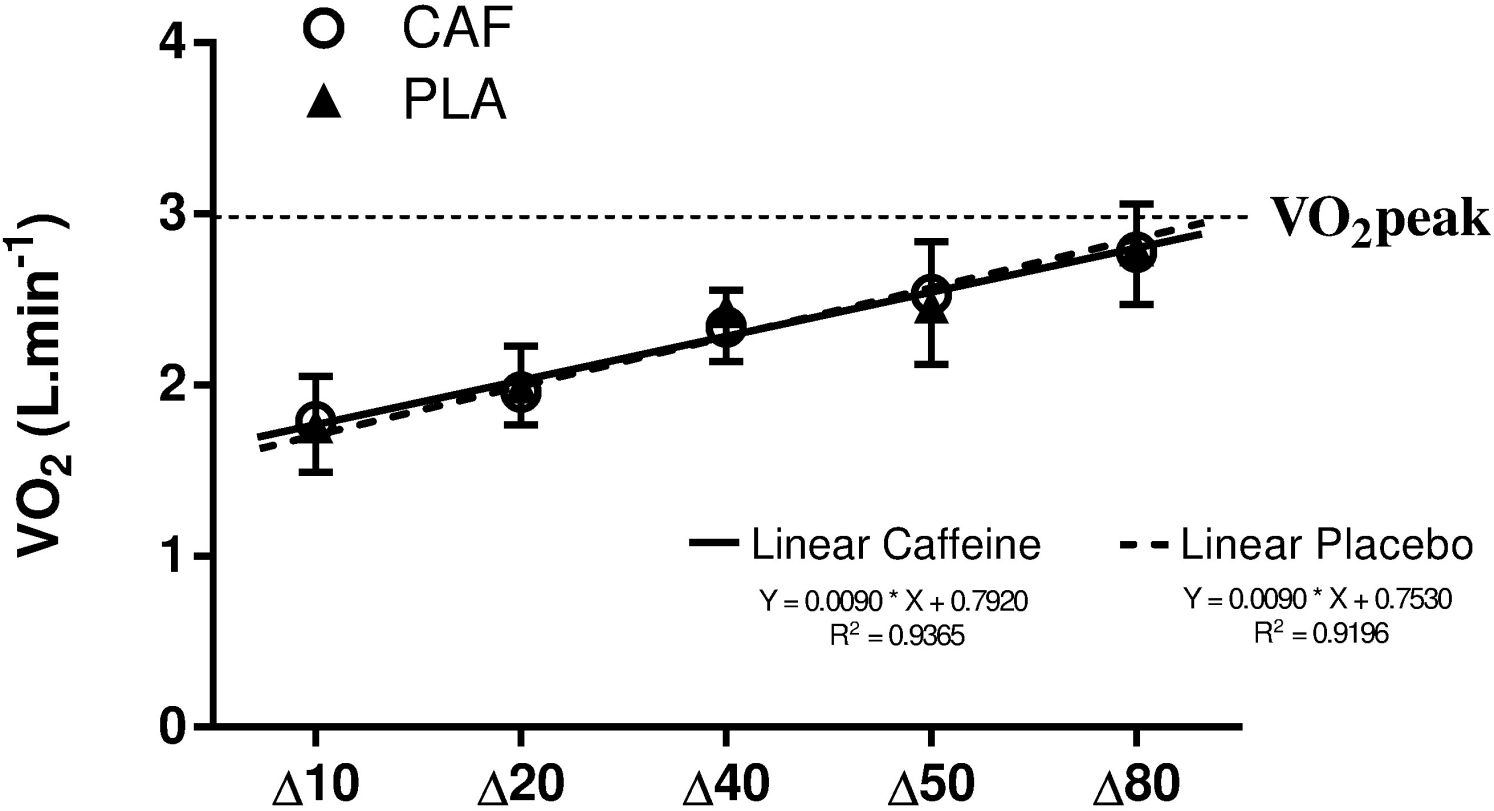
The VO_2_-power output relationship with caffeine (CAF) or placebo (PLA) (mean ± SD). Dashed horizontal line represents average VO_2_peak obtained during the incremental test. Continuous line represents fitted regression to caffeine and dashed line fitted regression to placebo. The regression line was used to estimate supramaximal oxygen demand.

**Fig 3:**
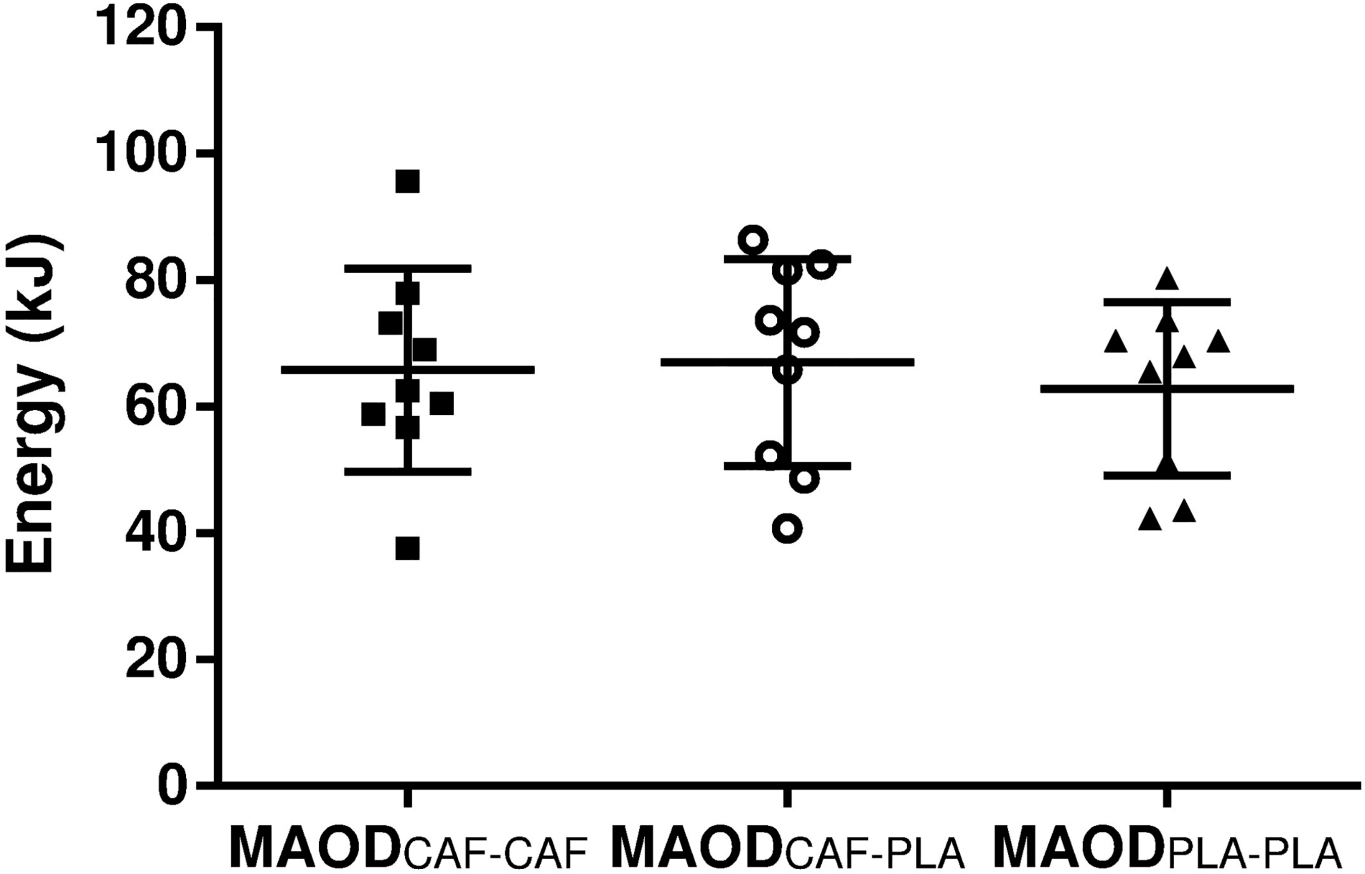
Effect of caffeine ingested before the supramaximal test on MAOD when measured by using estimated supramaximal oxygen demand with or without caffeine influence (mean ± SD). Closed squares (MAOD_CAF-CAF_), open circles (MAOD_CAF-PLA_) and closed triangles (MAOD_PLA-PLA_) represent individual values. MAOD_CAF-CAF_: maximal accumulated oxygen deficit with caffeine and demand-estimated with caffeine. MAOD_CAF-PLA_: maximal accumulated oxygen deficit with caffeine and demand-estimated without caffeine. MAOD_PLA-PLA_: maximal accumulated oxygen deficit with placebo and demand-estimated without caffeine.

Submaximal VO_2_ increased with the exercise intensity under both conditions (*P* < 0.01) but there was no difference between caffeine and placebo (*P* = 0.63) or interaction between intensity and condition (*P* = 0.12) (Fig 2). The strength of the submaximal VO_2_-power output regression (i.e., R^2^) was similar between caffeine and placebo (0.94 ± 0.04 and 0.92 ± 0.04, respectively, *P* = 0.38). The VO_2_-power output regression of each participant is reported as supplementary data (S1 Fig). The estimated oxygen demand was also similar between caffeine and placebo (7.88 ± 1.56 and 7.89 ± 1.30 L, respectively, *P* = 0.99). The accumulated VO_2_ during the supramaximal test was similar between caffeine and placebo (4.38 ± 0.90 and 4.54 ± 0.74 L, respectively, *P* = 0.14). Consequently, there was no difference between MAOD_CAF-CAF_ and MAOD_PLA-PLA_ (*P* = 0.99, Fig 3). When MAOD was calculated from submaximal tests without caffeine effect (i.e., MAOD_CAF-PLA_), there was also no difference between caffeine and placebo conditions (*P* = 0.41).

**Fig 4:**
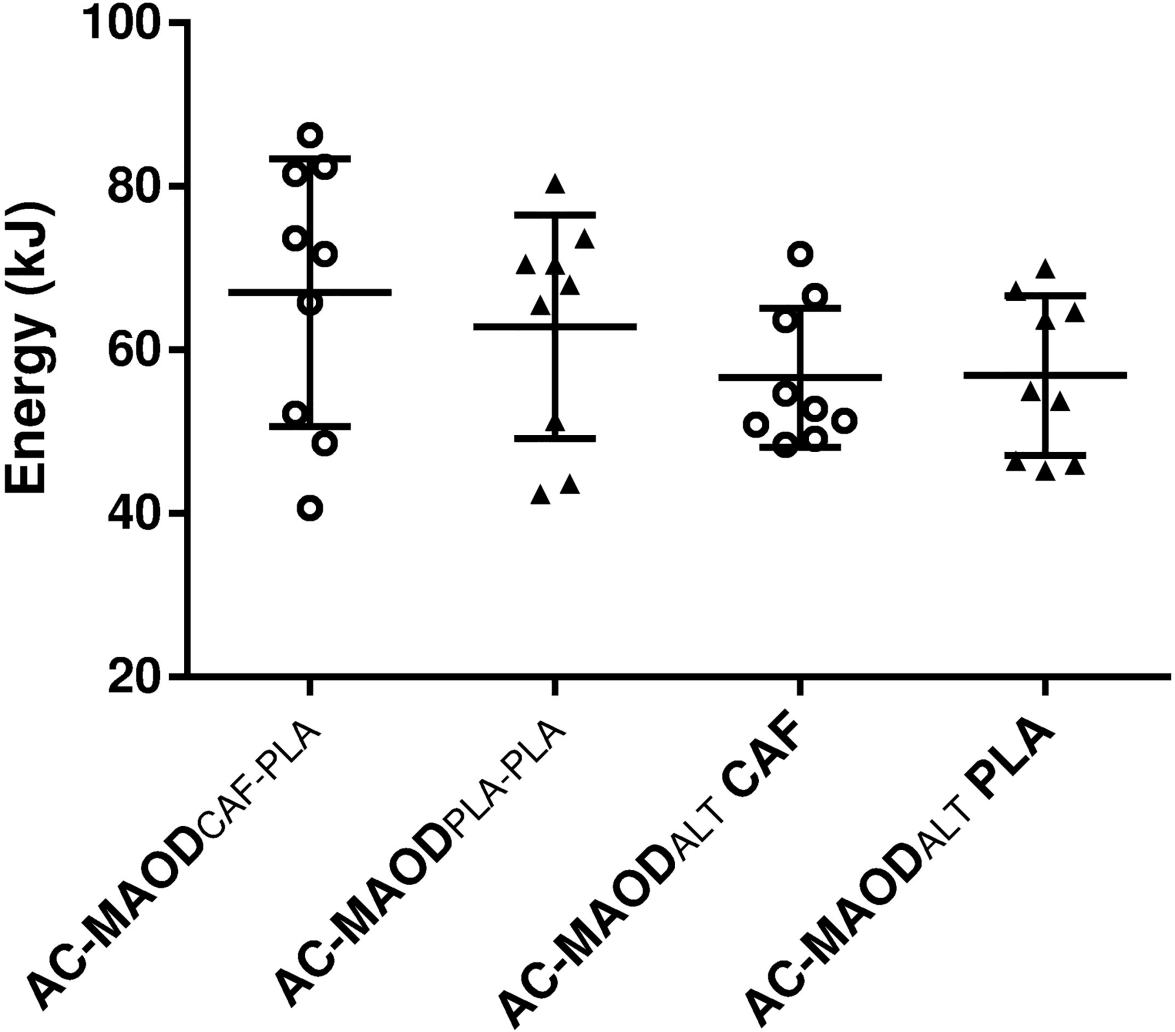
Comparison of anaerobic capacity (AC) measured with traditional and alternative maximal accumulated oxygen deficit methods (MAOD and MAODALT, respectively) after caffeine (CAF) or placebo (PLA) ingestion (mean ± SD). Open circles (CAF) and closed triangles (PLA) represent individual values.

There was no main effect of the condition or interaction for plasma [La^-^] response during the supramaximal bout (*P* = 0.40 and 0.79, respectively), but there was a main effect of time on plasma [La^-^], being greater post-exercise under both conditions (*P* < 0.01, Table 2). There was no difference between the effects of caffeine and placebo on plasma [La^-^] accumulation (*P* = 0.79, Table 2). Consequently, lactic component of MAOD_ALT_ was not significantly different between caffeine and placebo (38.1 ± 7.1 and 37.7 ± 7.1 kJ, *P* = 0.82, respectively). Similarly, A_1_ and τ_1_ were not significantly different under these conditions (*P* = 0.11 and 0.17, Table 2). Consequently, the alactic component of MAOD_ALT_ was not significantly different for either caffeine or placebo (18.4 ± 2.7 and 19.1 ± 5.1 kJ, *p* = 0.69, respectively). Traditional MAOD (MAOD_CAF-PLA_ and MAOD_PLA-PLA_) and MAOD_ALT_ (MAOD_ALT_ placebo and caffeine) were not significantly different from each other (*P* = 0. 07) (Fig 4). Caffeine had no effect on either method (*P* = 0. 17).

**Table 2:** Plasma lactate concentration and parameters of oxygen uptake recovery during a supramaximal exercise with caffeine and placebo influence (mean ± SD).

	Caffeine	Placebo
Pre-exercise [La^-^] (mmol·L^-1^)	1.5 ± 1.0	1.3 ± 0.6
Post-exercise [La^-^] (mmol·L^-1^)	9.9 ± 1.4^a^	9.5 ± 1.6^a^
Δ[La^-^] (mmol·L^-1^)	8.4 ± 1.6	8.3 ± 1.6
A_1_ (L·min^-1^)	1.0 ± 0.1	1.1 ± 0.1
τ_1_ (s)	54.9 ± 8.8	49.8 ± 8.5

[La^-^], plasma lactate concentration; Δ[La^-^], plasma lactate accumulation (post–pre-exercise); A_1_, amplitude of oxygen uptake recovery; τ_1_, time constant of oxygen uptake recovery

^a^ significantly higher than pre-exercise.

**Fig 5:**
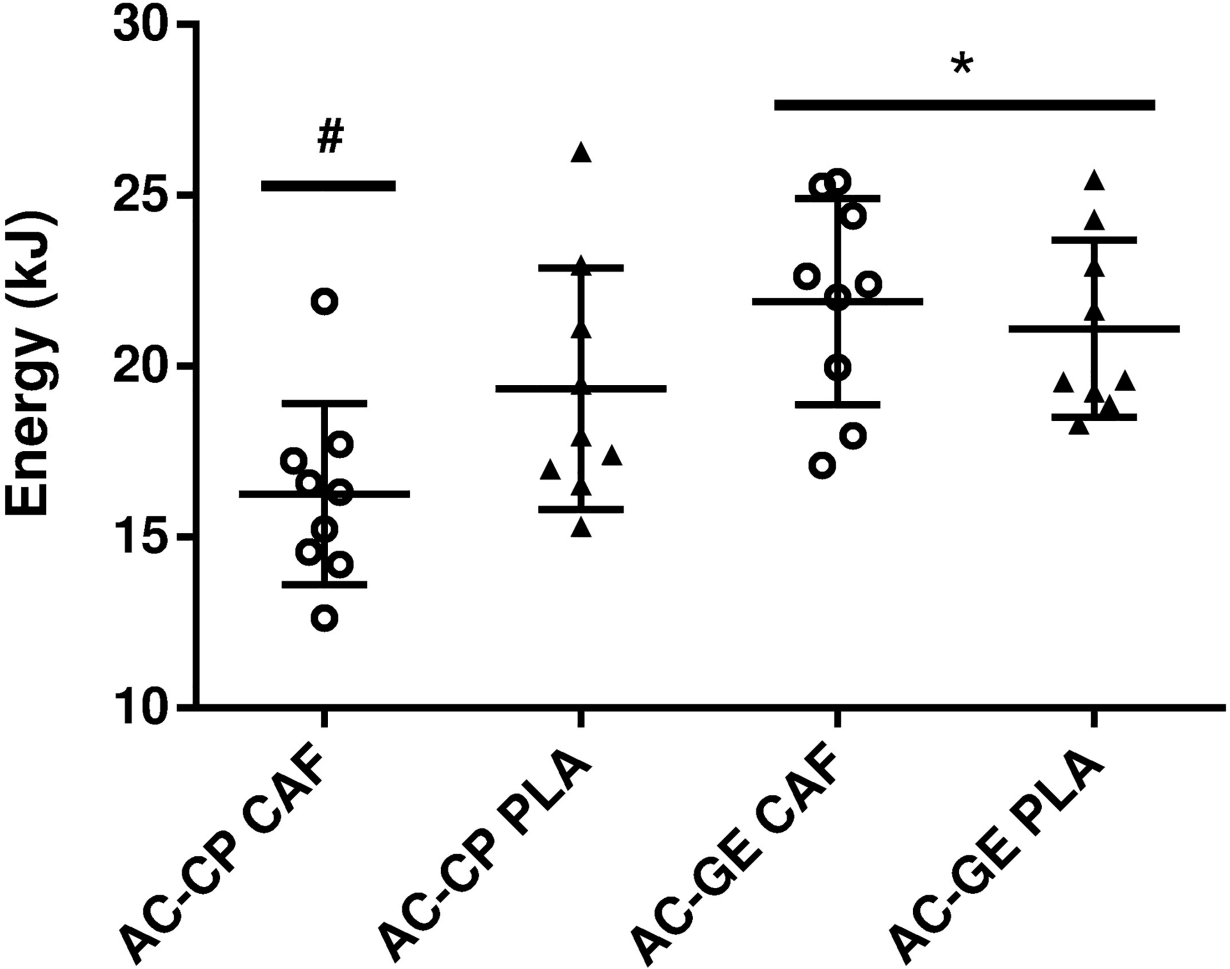
Comparison of the anaerobic capacity (AC) measured from critical power (CP) and gross efficiency (GE) methods after caffeine (CAF) or placebo (PLA) ingestion (mean ± SD). Open circles (CAF) and closed triangles (PLA) represent individual values. * Significantly higher than AC estimated from CP method (*P* < 0.01). # Significantly lower than AC-CP PLA (*P* < 0.01).

The CP linear inverse of time model (Eq 4) produced the lowest standard error of estimate under both caffeine and placebo conditions and was therefore used for further analysis. The AC estimated by GE method was significantly higher than the AC derived from CP method (*P* = 0.01, Fig 5). There was no main effect of condition (*P* = 0.21). However, there was a significant interaction between method and condition (*P* < 0.01); the AC was reduced with caffeine when estimated by the CP method, but not when estimated by the GE method. Contrarily, the CP values were higher in caffeine compared to the placebo (190 ± 27 and 165 ± 33 W, respectively; *P* = 0.01).

## Discussion

In the present study we demonstrated that caffeine ingestion during submaximal exercise bouts did not affect the estimate of the supramaximal oxygen demand and consequently, the estimation of the MAOD. Caffeine had no effect on AC, except when estimated by the CP method. In addition, the AC estimated by the CP method was lower than AC estimated by GE method.

A first interesting result of the present study was that caffeine did not increase the rate of increase of the VO_2_ with respect to exercise intensity (Fig 2). The submaximal exercise intensities used in the present study were within “heavy” (∆10–50) and “severe” (∆80) exercise intensities [41]. All these exercise intensities develop a VO_2_ slow component [42]. Caffeine has been reported to reduce the slow component of VO_2_ in the severe domain [20], although some studies have found no effect of caffeine on submaximal VO_2_ [18, 19]. Our results corroborate the studies showing no effect of caffeine on submaximal VO_2_ and ultimately suggest that caffeine ingestion before submaximal bouts does not affect the supramaximal oxygen demand estimate. Further, this result provides support for studies investigating the effect of caffeine on MAOD without the participant ingesting caffeine during the submaximal bouts [2, 7].

The MAOD_ALT_ has been proposed as a lesser time-consuming method, providing a similar estimate to the traditional MAOD [1]. Posterior investigation provided additional evidence of test and retest reliability of MAOD_ALT_ [43]. A recent study found that caffeine increased time to exhaustion at 115% of VO_2_peak, but the MAOD_ALT_ had not increased [26]. The authors suggested that caffeine promotes improvement in exercise tolerance by its action on the central nervous system rather than by an increase in AC. Our results add that MAOD_ALT_ accompanies traditional MAOD calculated without caffeine influence during submaximal bouts, detecting no effect of caffeine on AC (Fig 4). The reason for this is not fully clear, but may be related to the fact that the MAOD_ALT_ does not need an estimate of supramaximal oxygen demand compared to traditional MAOD. In fact, the supramaximal oxygen demand, and consequently MAOD, can be substantially influenced by the number, intensity, and duration of the submaximal exercise bouts [4]. In addition, an important advantage of the MAOD_ALT_ is that it provides a separate estimate of alactic and lactic components of the AC. Any effect of caffeine on anaerobic metabolism would be expected to be on the lactic metabolism. It has been speculated that caffeine might withdraw adenosine-mediated inhibition of phosphofructokinase, increasing the anaerobic glycolysis [12]. However, similar to what has been previously noticed [26], the results of the present study suggest that caffeine has a minimal influence on the maximal capacity to produce work involving the glycolytic energy system.

An expected effect of caffeine on AC was also undetectable using the GE method. However, the estimated AC by the GE method was ~ 20% higher compared to that estimated by *W´* (Fig 5). Because the GE and CP methods estimate AC using mechanical parameters, while the MAODs estimate AC using metabolic parameters, they cannot be interchangeably compared, at least if MAOD and MAOD_ALT_ have been corrected by individual gross efficiency. However, this procedure involves a “circular reasoning”, because one parameter involved in determining of AC by the GE method would be a direct correction of the AC determined by MAODs. The lack of a universally accepted method to measure AC makes it difficult to ascertain if the AC is either systematically overestimated by the GE method, or if it is underestimated by *W´*, or both. However, two important assumptions of the GE method must be taken into account. The rationale of this method assumes that gross efficiency remains similar between submaximal and supramaximal exercises, and also remains constant throughout the supramaximal exercise bout [5]. However, although the efficiency of the anaerobic metabolism is higher than that of the aerobic metabolism during high-intensity exercise [44], gross efficiency (calculated as the sum of the rates of anaerobic and aerobic ATP production divided by external work) might be reduced during supramaximal exercise compared to submaximal exercise, and also reduced over time during the supramaximal bout [45, 46]. In addition, when gross efficiency might be underestimated at exercise intensities lower than GET, and, thus, AC overestimated [5]. We calculated gross efficiency at 90% GET because the RER must be lower than 1.00 for precise determination of gross efficiency. Nevertheless, the GE method may be useful because it detected no differences between caffeine and placebo, as noted, using either MAOD or the MAOD_ALT_. However, if the intention is to compare the results with those reported in the literature or with absolute values used for the calculation of energy expenditure, this discrepancy has to be considered.

The AC estimated by *W’* was lower in caffeine than with the placebo (Fig 5). There is no reason to suspect that caffeine decreased AC because the time to exhaustion at the supramaximal bout was not reduced and this effect had not been noted when AC was estimated by the other methods. Interestingly, caffeine reduced the *W’* but increased the CP, an outcome similar to that reported related to the inspiration of hyperoxic gas (70% O_2_), which reduced the W′ and increased the CP [47]. This suggests that the CP and *W’* are interrelated and that the basic conceptual framework of the *W´* as a fixed anaerobic energy reserve must be reconsidered [36]. For this reconsideration, the magnitude of the *W´* might be attributed to the accumulation of fatigue-related metabolites, such as H^+^, Pi and extracellular K^+^ [36]. Thus, CP would represent a critical threshold for intramuscular metabolic control, above which exhaustive exercise results in non-metabolic stabilization, with the rate of accumulation increasing as a function of the power output above CP [36, 48]. The present study suggests that CP increased with caffeine, possibly increasing the critical threshold for metabolite accumulation over time, and in turn reducing the rate of metabolite accumulation for a given supra-CP exercise intensity. For example, ∆80 corresponded to ~ 139% of the CP estimated from the power-time curve with the placebo, but it only corresponded to ~ 120% of the CP estimated from the power-time curve with caffeine. Evidence showing a reduced rate of extracellular K^+^ accumulation with caffeine supports this assumption [27, 49].

Some limitations of the present study should be addressed. First, we were unable to confirm caffeine abstinence via analysis of blood caffeine concentration. However, an analysis of the food records confirmed that participants did not ingest any caffeine previous to the tests. In addition, previous findings have indicated that plasma caffeine levels rise to ~ 40 μmol·L^-1^, peaking at 60–90 min after ingestion when caffeine doses are administrated in capsules containing 5 mg·kg^-1^ body mass [28]. Secondly, plasma [La^-^] measured during exercise represents a balance between lactate production and clearance [50, 51]. This suggests that the anaerobic lactic contribution determined by the O_2_ equivalent from plasma [La^-^] may be influenced by lactate clearance, although lactate clearance may be reduced during high-intensity exercise. In addition, other O_2_ equivalents from blood/plasma [La^-^] rather than 3 ml O_2_·kg^-1^ body mass have been proposed [52]; and the assumption that EPOC_fast_ accounts only for PCr resynthesis is still under debated [53]. While these methodological issues may affect the estimate of MAOD_ALT_, they may have had a small effect in the present study because there was no difference between MAOD and MAOD_ALT_, and both methods indicated no effect of caffeine on AC. In addition, it should be recognized that recreationally active men were recruited for the present study (VO_2_peak ~ 41 mL·kg^-1^·min^-1^). Positive effect of caffeine on time to exhaustion during a supramaximal cycling bout has been reported in highly trained cyclists (VO_2_peak ~ 68 mL·kg^-1^·min^-1^) [27], or during supramaximal running bout in active athletes (VO_2_peak ~ 60 mL·kg^-1^·min^-1^) [2] and recreational runners (VO_2_peak ~ 56 mL·kg^-1^·min^-1^) [26]. However, using a similar sample of untrained participants (VO_2_peak ~ 43 mL·kg^-1^·min^-1^), Bell et al. [7] reported an improvement in time to exhaustion during a supramaximal cycling bout (125% VO_2_peak) with caffeine compared to the placebo. While it is unclear why we were not able to find a positive effect of caffeine on time to exhaustion during the supramaximal bout in the present study, we suppose that the level of training may account for the lack of effect of caffeine. It is important to note that it is very difficult to recruit highly trained athletes to participate in studies of this nature because of the number of visits involved, which could disrupt their training routines. Finally, determining VO_2_peak under caffeine influence could have provided further insights. However, as our main focus was to verify the effect of caffeine on anaerobic capacity, and participants had already visited the laboratory 11 times, we opted not to conduct one additional test. Nevertheless, this probably would have had a minimal impact on our results because caffeine had no effect on VO_2_ and time to exhaustion during the supramaximal test.

In conclusion, caffeine ingestion during submaximal bouts did not affect the VO_2_-power output curve, estimated supramaximal oxygen demand and MAOD. On the other hand, caffeine seems to have no detectable positive effect on AC; therefore the potential effect of caffeine on AC is questionable. Instead, the positive effect of caffeine on exercise endurance may be confined to submaximal exercise bouts. The *W´* derived from the CP concept may be not appropriate to reflect “anaerobic work capacity” as originally proposed.

## Supporting information

S1 Fig The VO_2_-power output relationship with caffeine (CAF) and placebo (PLA) for each participant. Continuous line represents fitted regression to caffeine and dashed line fitted regression to placebo. The regression line was used to estimate individual supramaximal oxygen demand.
(TIF)
