# Correction: Effect of caffeine ingestion on anaerobic capacity quantified by different methods

**DOI:** 10.1371/journal.pone.0183599

**Published:** 2017-08-15

**Authors:** Lucyana Arcoverde, Rodrigo Silveira, Fabiano Tomazini, André Sansonio, Romulo Bertuzzi, Adriano Eduardo Lima-Silva, Victor Amorim Andrade-Souza

The following information is missing from the Funding section: The page charge of this paper has been paid by PROEX-CAPES agreement.
