# Supplementary figures and images for: Effect of caffeine ingestion on anaerobic capacity quantified by different methods
Source: PLoS One. 2017 Jun 15;12(6):e0179457. doi: 10.1371/journal.pone.0179457 (PMC5472322; doi:10.1371/journal.pone.0179457)

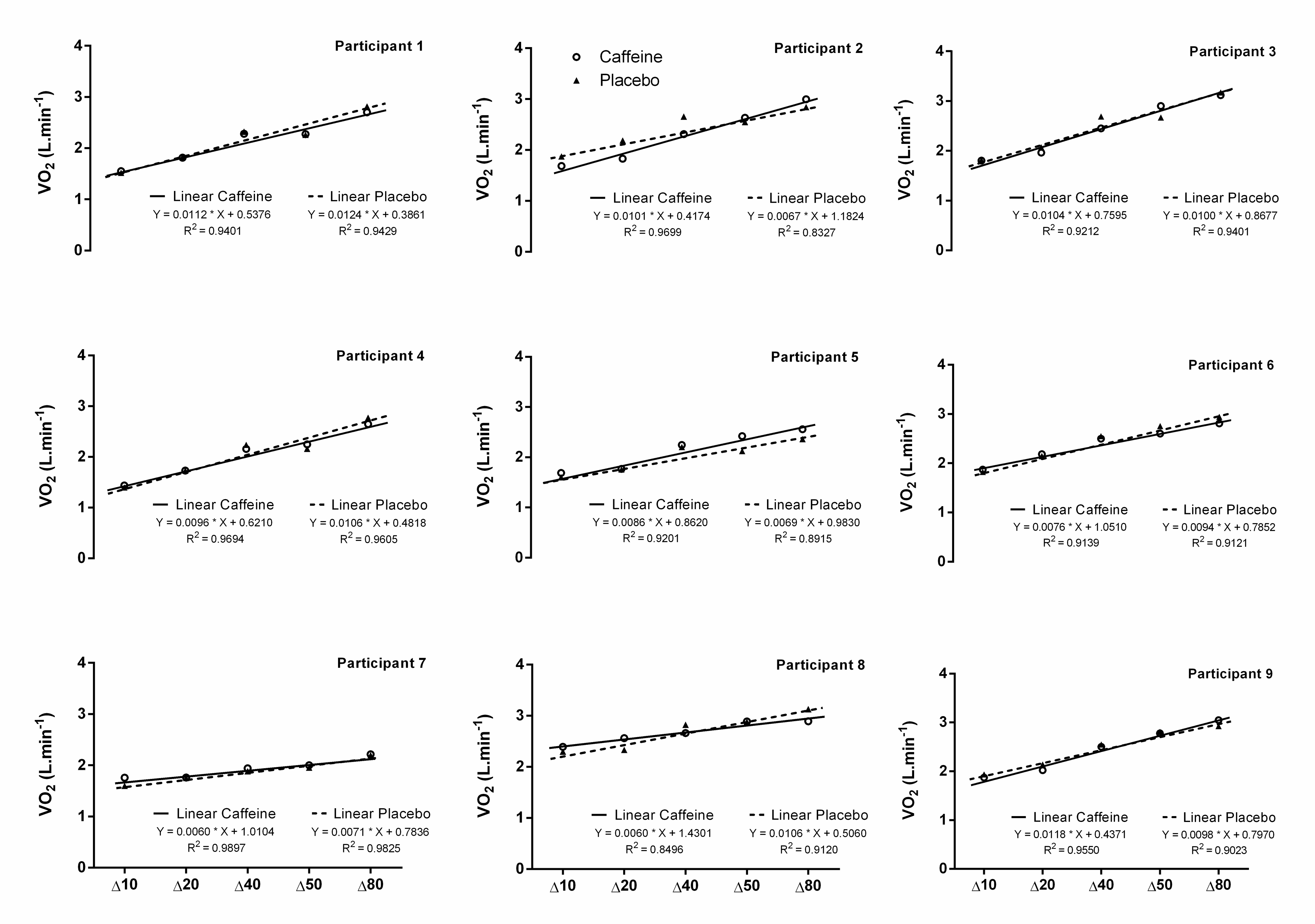

Supplement: S1 Fig — Continuous line represents fitted regression to caffeine and dashed line fitted regression to placebo. The regression line was used to estimate individual supramaximal oxygen demand. (TIF) [file pone.0179457.s001.tif]
